# Effects of dietary fat saturation level on growth performance, carcass traits, blood lipid parameters, tissue fatty acid composition and meat quality of finishing pigs

**DOI:** 10.5713/ajas.20.0247

**Published:** 2020-08-24

**Authors:** Jing Chen, Jiantao Li, Xianjun Liu, Yang He

**Affiliations:** 1 College of Animal Science & Veterinary Medicine, Shenyang Agricultural University, Shenyang 110866, China; 2 Shenyang Wise Diligence Agriculture-Technology Company Limited, Xinmin 110300, China

**Keywords:** Finishing Pig, Fatty Acid, Growth, Blood Lipid, Meat Quality

## Abstract

**Objective:**

The objective of this study was to investigate the effects of various dietary unsaturated to saturated fatty acids ratios (UFA to SFA ratios) on growth performance, carcass traits, blood lipid parameters, tissue fatty acid (FA) composition, and meat quality of finishing pigs.

**Methods:**

A total of 45 crossbred pigs ([Duroc×Landrace]×Yorkshire), with an average initial body weight of 60.3±2.4 kg, were randomly allocated to three treatment groups of 1:1, 2:1, and 3:1 dietary UFA to SFA ratios.

**Results:**

Both average daily gain and average daily feed intake of pigs were decreased linearly (p<0.05), whereas backfat thickness was decreased linearly (p<0.05) with increasing of dietary UFA to SFA ratio. Serum triglyceride and low density lipoprotein cholesterol were decreased quadratically or linearly (p<0.05) respectively, whereas high density lipoprotein cholesterol was increased quadratically (p<0.05) with increasing dietary UFA to SFA ratio. In M. longissimus thoracis, the proportion of C18:1 and monounsaturated FA was decreased linearly (p<0.05), whereas the proportion of C18:2n-6, C20:4n-6 and polyunsaturated FA (PUFA) were increased linearly (p<0.05) as dietary UFA to SFA ratio increased. In the subcutaneous adipose tissue, the proportion of SFA was decreased linearly (p<0.05), whereas the proportion of n-6 PUFA, n-3 PUFA, and the UFA to SFA ratios were increased linearly (p<0.05) with increasing of dietary UFA to SFA ratio. Meat color scores and shear force of pigs were decreased linearly (p<0.05), whereas drip loss and cooking loss were increased linearly (p<0.05) with increasing of dietary UFA to SFA ratio.

**Conclusion:**

Appropriately boosted dietary UFA to SFA ratio could be conductive to optimize blood lipid parameters and tissue FA composition. However, when the ratio is too high or too low it tends to have negative effects on growth performance and meat quality.

## INTRODUCTION

Fat has a high energy value, and is therefore used as a dietary supplementation to increase the energy density of feeds and thereby improve the energy status of animals and modify the fatty acids (FA) composition of animal tissues. There are a number of fat sources and fat combinations that are used to provide FA. Animal oils (with the exception of fish oil) and a few vegetable oils, such as palm oil, tend to contain higher proportion of saturated FA (SFA), while most vegetable oils tend to contain higher proportion of unsaturated FA (UFA). It is widely accepted that SFA and UFA have opposite effects on health. Studies have shown that consuming high quantities of SFA is associated with increased risks of obesity and related diseases such as insulin resistance, inflammation, hepatic steatosis, and cardiovascular diseases, whereas replacing SFA with UFA, including monounsaturated FA (MUFA) and polyunsaturated FA (PUFA), reduces the risk of coronary heart disease [1]. Therefore, nutritional guidelines for maintaining cardiovascular health are therefore gradually shifting from recommending overall reductions in saturated fat intakes towards improving the quality of the fat consumed. The 2013 Chinese Dietary Nutrient Intake (DRI) guidelines recommended that diets contain no more than 10% of their total energy content in the form of SFA [2]. However, Chinese SFA intakes may well be higher than this recommended value [3].

It is therefore clear that both the total fat content and the unsaturated to saturated FA ratio (UFA to SFA ratio) of the diet are highly important. Meat is an important source of fat and FA for humans. Although opinions differ on the importance of the FA composition for meat quality, consumers increasingly prefer meat products with higher proportion of UFA because of their beneficial effects on health [4]. There has consequently increasing interest in adjusting dietary UFA to SFA ratio to manipulate the FA composition of the meat, and thereby produce functional foods.

In monogastric animals, tissue FA composition and the FA endogenous synthesis are influenced by dietary FA composition. By providing UFA via the incorporation of UFA-rich oils into pig feed, it is possible to increase the deposition of UFA in the meat [5]. A number of previous studies have reported that the dietary supplementation of UFA-rich oils affects growth performance, meat quality and tissue FA composition [5,6]. However, relatively few studies have investigated the effects of the dietary UFA to SFA ratio on growth performance, carcass traits, or meat quality. Therefore, the objective of this study was to evaluate the effects of adding oil sources containing different UFA to SFA ratios on growth performance, carcass traits, blood lipid parameters, tissue FA composition, and meat quality in finishing pigs.

## MATERIALS AND METHODS

### Animal care

All procedures involving animals were reviewed and approved by the Institutional Animal Care and Use Committee of the College of Animal Science & Veterinary Medicine, Shenyang Agricultural University (SYXK (Liao) 2011 – 0001).

### Experimental animals and dietary treatments

A total of 45 crossbred pigs ([Duroc×Landrace])×Yorkshire] were assigned to three dietary treatments according to their initial body weights (60.3±2.4 kg), with five replicates per group and three pigs per replicate (two barrows and one gilt) in a completely randomized design. Three diets were formulated according to the NRC [7] nutrient requirements with UFA to SFA ratios of 1:1, 2:1, and 3:1 by manipulating the ratio of hydrogenated lard (Shuanghui Group Co., Ltd., Zhengzhou, Henan, China) to soybean oil (Jiuzhou Dadi biotechnology Co., Ltd., Liaoning, China). Diets were isonitrogenous (15.7% protein) and not isoenergetic (12.27, 12.30, 12.31 MJ ME/kg, respectively) (Tables 1, 2). Diets and oil were stored in a cool and dry workshop. The environment within the workshop was controlled using a thermostat and fan ventilation (the temperature remained between 10°C and 15°C, and the relative humidity remained between 50% and 55%) during the whole experiment. The pigs were housed in individual crates with concrete flooring and received feed and water *ad libitum*. The experiment lasted for 45 d.

### Sample collection

On day 44, blood was obtained via jugular vein puncture from two pigs (near average body weight) per replicate (one barrow and one gilt) and collected in 10 mL tubes. Serum was separated by centrifugation at 1,500×g at 4°C for 20 min and then stored at −20°C until analysis. The pigs were then fasted overnight and, on day 45, stunned electrically (240 V, 800 Hz for 5 to 6 s) and exsanguinated. The carcasses were subsequently split into two parts, and samples (circa 300 g) of the M. longissimus thoracis (LT) and subcutaneous fat were excised at the level of last rib from the right-hand sides of the carcasses and placed at 2°C for 24 h, after which the FA composition and meat quality were analyzed.

### Analytical methods

#### Growth performance and carcass traits

Body weights were recorded in the morning before feed distribution from the start of the trial until a final average live weight of 100.2±4.4 kg, and the feed intake was recorded weekly. The average daily gain (ADG), average daily feed intake (ADFI), and gain:feed were calculated. After evisceration, the carcasses were weighed to obtain the carcass weights. The loin eye area was measured by tracing the LT surface at the 10th, and the backfat thickness measurements were obtained at the level of last rib using a real-time ultrasound instrument (Piglog 105, SFK Technology, Herlev, Denmark).

#### Serum biochemical analyses

The serum total cholesterol (TC) concentration was determined spectrophotometrically, while the triglyceride (TG) concentration was determined using the GPO-PAP enzymatic assay. The high density lipoprotein cholesterol (HDL-c) concentration was determined using the phosphotungstic acid- magnesium precipitation method, and the low density lipoprotein cholesterol (LDL-c) concentration was determined using the polyethylene sulfuric acid precipitation assay. Serum TC, TG, HDL-c, and LDL-c concentrations were measured by using an automatic biochemistry analyzer (Hitachi 747, Hitachi Ltd., Tokyo, Japan) with a commercially available kits (Jiangsu Baolai Bio-Technology Co., LTD., Yancheng, China), according to the manufacturers’ instructions.

#### Fatty acid composition

The FA compositions of the feed, the LT and subcutaneous fat samples were determined using gas chromatography. Approximately 5 g samples were extracted using a mixture of chloroform and methanol (v/v 2:1), and FA methyl esters were obtained using the ISO 5509 method. After phase separation, the upper layer was retained, and a 2 mL aliquot was transferred to a sample injection bottle. The FA composition of the sample was then measured using capillary gas chromatography, with separation by a J&W DB-23 (65.0 m×250 μm×0.25 μm film thickness) fused silica capillary column (Supelco, Sigma-Aldrich, St. Louis, MO, USA), installed on an Agilent 7820 A gas chromatograph (Agilent Technologies, Inc., Palo Alto, CA, USA) equipped with a flame ionization detector. To optimize separation, the initial oven temperature was set at 180°C and held for 10 min, then increased by 4°C/min to 200°C and held for 15 min, and finally increased by 10°C/min to 230°C and held for 6 min. Helium was used as the carrier gas, with a flow rate of 24 cm/s. Both the injector and detector were set at 250°C and the split ratio was 20:1. Peaks were identified using purified standards, and FA was identified by comparing their relative FA methyl ester peak retention times to those of the standards. The FA composition was calculated by relating individual FA to the sum of all the FA detected.

#### Meat quality

At 45 min post-mortem, the initial pH (pH_45_) was directly measured at the level of last rib using a pH meter (Model AR25, Fisher Scientific, Pittsburgh, PA, USA). The ultimate pH (pH_24 h_) was measured at 24 h post-mortem. The sensory evaluation for visual color was measured using a five-point scoring system according to NPPC (2000) standards at an ambient temperature of 25°C on LT sample surface at 1 h post-mortem. The drip loss was measured using approximately 4.5 g meat samples according to the plastic bag method [6]. To evaluate the cooking loss and shear force, a slice (200±20 g) cut from each LT chop was weighed, placed in a plastic bag and cooked to an internal temperature of 70°C in an 80°C water bath for 10 min. The cooked samples were allowed to cool at room temperature (25°C) for 30 min, blotted dry and weighed. The samples were then cut parallel to the long axis of the muscle fibers into 30 mm long, 10×10 mm cross-section rectangular slices. The shear force was determined using a texture analyzer (QTS25, Brookfield, NY, USA). Each slice was sheared at a constant speed of 0.5 mm/s [8].

### Statistical analysis

All data were analyzed by one-way analysis of variance using SPSS version 19.0 for Windows (SPSS Inc., Chicago, IL, USA). Polynominal contrasts (linear or quadratic) were conducted to evaluate the effect of UFA to SFA ratio. Probability values <0.05 were considered significant. The results are presented as mean values with their standard errors. For growth performance, carcass traits, tissue FA composition and meat quality, replicate was used as experimental unit, while for blood analysis the individual pig was used as the experimental unit.

## RESULTS

### Growth performance and carcass traits

Both ADG and ADFI of pigs were decreased linearly (p<0.05) with increasing of dietary UFA to SFA ratio (Table 3). Backfat thickness was decreased linearly (p<0.05) with increasing of dietary UFA to SFA ratio. The different dietary UFA to SFA ratios had no effect on gain:feed, carcass yield (as a percentage of the live weight) or loin eye area.

### Serum lipid parameters

Serum TG concentration was decreased quadratically (p<0.05) and LDL-c concentration was decreased linearly (p<0.05), while HDL-c concentration was increased quadratically (p< 0.05) with increasing of dietary UFA to SFA ratio (Table 4). There were no differences in serum TC concentration among the dietary treatments.

### Tissue fatty acid composition

In the LT, the proportion of C18:1 was decreased linearly (p<0.05), whereas the proportion of C18:2n-6 and C20:4n-6 were increased linearly (p<0.05) as dietary UFA to SFA ratio increasing from 1:1 to 3:1 (Table 5). Furthermore, the proportion of MUFA was decreased linearly (p<0.05), whereas the proportion of PUFA was increased linearly (p<0.05) with increasing of dietary UFA to SFA ratio. The different dietary UFA to SFA ratios had no effects on the proportion of UFA, SFA, and the UFA to SFA ratio.

In the subcutaneous adipose tissue, the proportion of C16:1 and C20:4n-6 were decreased quadratically (p<0.05) and the proportion of C18:0, C18:1, C20:0 and C20:1 were decreased linearly (p<0.05) with increasing of dietary UFA to SFA ratio (Table 6). However, the proportion of C14:1 was increased quadratically (p<0.05) and the proportion of C18:2n-6, C18:3n-3, and C20:3n-6 were increased linearly (p<0.05) as UFA to SFA ratio increased. In addition, the proportion of PUFA was increased linearly (p<0.05) as UFA to SFA ratio increased, while the opposite trend (p<0.05) was found for the SFA. In contrast with the lack of change in the UFA to SFA ratio in the LT, the UFA to SFA ratio in the adipose tissue increased linearly (p<0.05) with an increase in the dietary UFA to SFA ratio.

### Meat quality

Meat color score and shear force of pigs were decreased linearly (p<0.05), whereas drip loss and cooking loss were increased linearly (p<0.05) with increasing of dietary UFA to SFA ratio (Table 7). However, the different dietary UFA to SFA ratios had no significant effects on meat pH (at 45 min and 24 h).

## DISCUSSION

### Effects of dietary UFA to SFA ratios on growth performance and carcass traits

Previous studies have shown that replacing part of the metabolizable energy content of a diet with different fat mixtures does not change the energy content or nutritional value of the diet and, consequently have no effect on growth performance and carcass quality of finishing pigs [5,8,9]. Some studies reported that the digestibility of dietary fats increases when the diet contains a greater proportion of UFA, which may increase the energy digestibility of the diet and thus improve growth performance. This is consistent with the reports, in which pigs fed 5% soybean oil had higher ADG than pigs fed 5% choice-grade white grease or beef tallow [10]. However, in this study, both ADG and ADFI were higher in the pigs fed the diet with lower UFA to SFA ratio, which suggests that pigs fed diet with lower UFA:SFA tend to grow faster. It may be due to the fact that lard-derived SFA tends to be deposited in fat tissue depots, whereas soybean oil-derived UFA tends to be oxidized preferentially over SFA for energy production, and therefore decrease ADFI. This results concurred with a previous study, in which there were significantly increase in ADG and ADFI of barrows as UFA to SFA ratio increased. Interestingly, the opposite effect has been observed in gilts [8]. The generally conflicting reports on the effects of the dietary FA composition on growth performance of pigs may be due to differences in the diet compositions, energy content, fat supplementation levels tested, the genotypes or genders of the pigs used, as well as number of experiment unit involved.

The dietary UFA to SFA ratios in this study did not affect the carcass yields or loin eye area, but affected the backfat thickness, with lower backfat thickness being observed in the pigs fed the diet with UFA to SFA ratio of 3:1. This result concurred with the observation that pigs fed a diet supplemented with soybean oil tended to have lower backfat thickness than pigs fed a diet supplemented with palm oil [11]. These results suggested that the dietary fat saturation level affected the degree of fat deposition, with a lower fat saturation resulting in thinner backfat [12,13]. Soybean oil contains greater concentration of UFA than most animal fat used in commercial swine diets. Dietary UFA are the most effective inhibitors of *de novo* fat synthesis [14], with the more unsaturated the dietary fat, the lower the fat deposition. The mechanisms underlying the effects of fat saturation level on fat deposition rates in pigs are still unclear, although it may be related to the preferential mitochondrial transport and β-oxidation of UFA rather than SFA [15].

### Effects of dietary UFA to SFA ratios on serum lipid parameters

It has been recognized that lower blood TG, TC, and LDL-c levels, and higher blood HDL-c level, are beneficial for the health of humans and animals [6]. In this study, the pigs fed the diets with UFA to SFA ratios of 2:1 and 3:1 had lower TG and LDL-c concentrations and a higher HDL-c concentration in serum than the pigs fed the diet with UFA to SFA ratio of 1:1. Soybean oil-derived UFA increased HDL-c concnetration and decreased TG concnetration by increasing synthesis of HDL and clearance of TG. However, SFA elevated serum LDL-c levels part by decreasing apolipoprotein B (apoB/E) receptor-mediated fractional LDL turnover and increasing LDL-c production rates [16]. Similarly, pigs fed diets containing corn oil or linseed oil had lower blood LDL-c and TG, and higher HDL-c than pigs fed diets containing lard, beef tallow, lauric acid, myristic acid, or palm oil [17–19].

### Effects of dietary UFA to SFA ratios on tissue fatty acid composition

Soybean oil contains high levels of C18:2n-6 and moderate levels of C18:1 and C18:3n-3. As the essential FA, C18:2n-6, and C18:3n-3 are incorporated directly into the tissue lipids or are converted into their derivative n-6 or n-3 PUFA, respectively, whereas SFA such as C14:0, C16:0 and C18:0, and MUFA such as C16:1 and C18:1, may be *de novo* synthesized [20]. It is therefore likely that high proportion of dietary PUFA are incorporated into the body fat than dietary SFA and MUFA. The results of this study therefore concurred with expectations, with the proportion of total PUFA and n-6 PUFA, including C18:2n-6 and C20:4n-6, being markedly increased in the LT of the pigs fed the soybean oil diet, whereas the proportion of MUFA, such as C18:1, were reduced accordingly, although there were no effects on the proportion of SFA or on the UFA to SFA ratio. This was in agreement with previous reports that pigs fed diets containing soybean oil or sunflower oil had higher proportion of n-6 PUFA in their meat than those fed diets containing palm oil, hydrogenated lard, or choice-grade white grease [12,21]. In the subcutaneous adipose tissue, the diet with UFA to SFA ratio of 3:1 increased the proportion of C18:2n-6 and C18:3n-3, and decreased the proportion of SFA, and the UFA to SFA ratio. The similar findings were also reported in pigs fed linseed oil and sunflower oil when compared to pigs fed beef tallow or lard [9,21,22].

In regard to the FA deposition in the different tissues, this study found that SFA and MUFA were the predominant components in the LT and subcutaneous adipose tissue, and the proportion of PUFA was relatively low. However, when we compared the different tissues of pigs fed with the same diet, the FA deposition rate appeared to be less consistent. For example, the proportion of C18:3n-3 was higher in the LT than in the subcutaneous adipose tissue when pigs were fed the same diet, whereas the proportion of C18:2n-6 was higher in the subcutaneous adipose than in the LT, which suggests that FA may have different incorporation rates in different target tissues. Furthermore, we observed that the subcutaneous adipose tissue contained more types of FA than the LT did, which indicates that the effects of the dietary fat saturation level on the FA composition of the adipose tissue were greater than that on the LT. This may have been because of the higher lipid content of the subcutaneous adipose tissue, and therefore its higher capacity for lipogenesis than other tissue types, including muscle tissue [23]. In addition, the fat content of the LT is relatively stable in finishing pigs, whereas that of adipose tissue increases steadily throughout this growth phase, suggesting that the subcutaneous adipose tissue may be more susceptible to changes in the dietary FA composition [24].

### Effects of dietary UFA to SFA ratios on meat quality

The meat quality evaluation included measurements of the pH, color, shear force, cooking loss, and drip loss. In this study, the dietary fat saturation level had no effect on the pH_45_ or the pH_24 h_, which concurred with the results of previous studies [8,25]. However, the pH_45_ and pH_24 h_ values recorded for all the treatment groups in this study were very close to the thresholds for normal meat (6.5>pH_45_>6.0; pH_24 h_>5.5). This contrasted with the results of other studies, in which the authors observed pH_24_ values below 5.5, indicating that the pork tended to be pale, soft, and exudative [5,26]. In general, there are correlations between the pH and other meat quality indicators, with an decrease in the pH_24_ being associated with paler meat, increased cooking loss and decreased shear force values.

Meat color is a sensory indicator for meat freshness, and directly related to the content of myoglobin. The lower scores the paler the meat is, the higher scores the darker the meat is, and with 3 score being desirable. In this study, muscle color was significantly affected by the dietary FA composition, declining with an increase in the dietary UFA to SFA ratio. Pigs fed the diet with UFA to SFA ratio of 2:1 had a color score close to normal (3 score), while pigs fed the diet with UFA to SFA ratio of 3:1 had the lowest color score (2.5). The lower oxidative stability of soybean oil-derived UFA may result in the increased occurrence of lipid oxidation, and as the oxidation of fat is related to the formation of precursors of oxymyoglobin oxidation, and thus to the formation of metmyoglobin, this may result in a decrease in meat redness [27].

Studies have shown that feeding more unsaturated oil to pigs could negatively impact the processing quality of the carcass fat because of the reduction of the fat melting point [13], whereas more dietary SFA could favor meat quality for processing because of its higher melting point and better oxidative stability [21,28,29]. Tenderness is an important indicator as evaluating meat eating quality and reflects the fat content of the muscle to a certain extent. One objective measuere of the tenderness is the force required to shear a piece of meat. In this study, the shear force was the lowest in the pigs fed the diet with UFA to SFA ratio of 3:1. Furthermore, the pigs fed the diet with UFA to SFA ratio of 3:1 appear to have an undesirable water loss with regard to the drip loss and cooking loss, which suggests that the dietary supplementation with the soybean oil-derived UFA has a negative effect on its water-holding capacity. Dietary supplementation with UFA tends to induce the rapid oxidation of carcass fat [11,30], which could result in the development of undesirable carcass characteristics such as soft fat and low meat water-holding capacity, and these negative changes tend to get worse with the increase in the amount of UFA supplementation [8]. Therefore, further studies should be performed to define the optimum UFA to SFA ratio and fat supplementation level to limit the risks of increasing lipid oxidation and having adverse effects on product quality.

In conclusion, the dietary UFA to SFA ratio affects growth performance, serum lipid parameters, and meat quality of finishing pigs. Increasing UFA to SFA ratio appear to have beneficial effects on the serum lipid parameters and tissue FA deposition, with negative effects on growth performance and meat quality to some extent. Although the health benefits of UFA are well known, there are still concerns about their susceptibility to lipid oxidation. Further research into the optimum dietary UFA to SFA ratio and the mechanisms responsible for the effects of dietary fat composition on growth performance and meat quality is therefore necessary.

## Figures and Tables

**Table 1:** Composition and analysis of experimental diets (air-dry basis)

Item	UFA to SFA ratio		

	1:1	2:1	3:1
Ingredients (%)			
Corn	56.70	56.70	56.70
Soybean meal (47.5% CP)	16.00	16.00	16.00
Distillers dried grains with solubles	3.00	3.00	3.00
Wheat middlings	10.00	10.00	10.00
Rice bran meal	5.00	5.00	5.00
Cottonseed meal	2.00	2.00	2.00
Limestone	1.20	1.20	1.20
Dicalcium phosphate	0.40	0.40	0.40
Salt	0.20	0.20	0.20
Sodium bicarbonate	0.20	0.20	0.20
Hydrogenated lard	2.00	0.80	0.00
Soyabean oil^1)^	0.00	1.20	2.00
Premix^2)^	3.30	3.30	3.30
Analyzed composition (%)			
ME (MJ/kg)^3)^	12.27	12.30	12.31
CP	15.70	15.70	15.70
Crude lipid	4.70	4.70	4.70
Ca	0.60	0.60	0.60
Avaliable P	0.20	0.20	0.20
Lysine-HCl	0.90	0.90	0.90
Methionine+cystine	0.80	0.80	0.80
Threonine	0.60	0.60	0.60
Tryptophan	0.20	0.20	0.20

UFA, unsaturated fatty acids ratio; SFA, saturated fatty acids ratio; CP, crude protein; ME, mebolizable energy.

1) To replace equivalent amounts of lard, 0.0%, 1.2%, and 2.0% of soyabean oil were used, making the dietary UFA to SFA ratios about 1:1, 2:1, and 3:1, respectively (Table 2).

2) Premix provided per kg diet: retinol acetate, 6,500 IU; thiamin, 0.34 mg; riboflavin, 4.23 mg; pyridoxine, 1.76 mg; cobalamin, 17 μg; cholecalciferol, 1,710 IU; DL-α-tocopherol acetate, 12.8 IU; menadione, 1.32 mg; nicotinic acid, 26.45 mg; pantothenic acid, 10.57 mg; biotin, 0.22 mg; folic acid, 0.88 mg; Cu (as CuSO_4_·5H_2_O), 20 mg; Fe (as FeSO_4_·7H_2_O), 60 mg; Zn (as ZnSO_4_·7H_2_O), 80 mg; Mn (as MnSO_4_·H_2_O), 15 mg; I (as KI), 1.5 mg; Se (as Na_2_SeO_3_·5H_2_O), 0.45 mg.

3) ME is calculated value.

**Table 2:** Fatty acid composition of the experimental diets (% total fatty acids)

Fatty acid	UFA to SFA ratio		

	1:1	2:1	3:1
C10:0	-	-	0.24
C12:0	0.45	0.33	0.24
C14:0	17.03	12.49	9.70
C14:1	0.18	0.44	0.60
C16:0	19.54	16.23	11.49
C17:0	0.97	0.69	0.52
C18:0	6.45	3.85	2.72
C18:1	38.95	32.42	25.68
C18:2 n-6	15.67	32.60	47.28
C18:3 n-3	0.11	0.47	1.22
C20:0	0.63	0.49	0.31
MUFA	39.13	32.86	26.28
PUFA	15.78	33.06	48.50
UFA	54.91	65.92	74.77
SFA	45.09	34.10	25.23
UFA:SFA	1.22	1.93	2.96

UFA, unsaturated fatty acids; SFA, saturated fatty acids; MUFA, monounsaturated fatty acid (the sum of C14:1, C18:1); PUFA, polyunsaturated fatty acid (the sum of C18:2 n-6, C18:3 n-3); UFA, unsaturated fatty acid (the sum of MUFA, PUFA); SFA, saturated fatty acid (the sum of C10:0, C12:0, C14:0, C16:0, C17:0, C18:0, C20:0).

**Table 3:** Effects of dietary unsaturated to saturated fatty acids ratios on growth performance of finishing pigs

Items	UFA to SFA ratio			SEM (n = 5)	p-value	
	
	1:1	2:1	3:1		Linear	Quadratic
ADG (kg/d)	0.94	0.85	0.81	0.02	0.002	0.778
ADFI (kg/d)	2.43	2.31	2.18	0.04	0.001	0.554
Gain:feed	0.39	0.37	0.37	0.01	0.330	0.555
Carcass yield (%)	71.76	71.62	71.79	0.33	0.979	0.933
Backfat thickness (mm)	16.18	15.47	15.32	0.08	<0.001	0.066
Loin eye area (cm^2^)	51.85	51.45	51.43	0.13	0.057	0.373

UFA, unsaturated fatty acids; SFA, saturated fatty acids; SEM, standard error of the mean; ADG, average daily gain; ADFI, average daily feed intake; LT, M. longissimus thoracis.

**Table 4:** Effects of dietary unsaturated to saturated fatty acids ratios on serum lipid parameters of finishing pigs

Items	UFA to SFA ratio			SEM (n = 10)	p-value	
	
	1:1	2:1	3:1		Linear	Quadratic
TG (mmol/L)	1.44	1.35	1.36	0.018	0.398	0.008
TC (mmol/L)	3.95	3.80	3.86	0.088	0.334	0.385
HDL-c (mmol/L)	1.42	1.54	1.53	0.021	0.381	0.050
LDL-c (mmol/L)	2.38	2.31	2.26	0.018	0.018	0.063

UFA, unsaturated fatty acids; SFA, saturated fatty acids; SEM, standard error of the mean; TG, triglyceride; TC, total cholesterol; HDL-c, high density lipoprotein cholesterol; LDL-c, low density lipoprotein cholesterol.

**Table 5:** Effects of dietary unsaturated to saturated fatty acids ratios on fatty acid composition of the LT (% total fatty acids)

Items	UFA to SFA ratio			SEM (n = 5)	p-value	
	
	1:1	2:1	3:1		Linear	Quadratic
C10:0	0.03	0.03	0.03	0.003	0.506	0.540
C14:0	0.80	0.87	0.87	0.032	0.047	0.097
C16:0	23.66	23.40	23.16	0.133	0.198	0.440
C17:0	0.05	0.05	0.04	0.006	0.145	0.189
C18:0	18.94	18.60	18.65	0.242	0.263	0.433
C18:1n-9	37.37	35.72	28.63	0.454	<0.001	0.070
C18:2n-6	11.59	13.59	20.68	0.372	<0.001	0.052
C18:3n-3	5.40	5.40	5.60	0.114	0.167	0.226
C20:0	0.03	0.01	0.02	0.004	0.112	0.063
C20:4n-6	2.13	2.32	2.31	0.053	0.014	0.173
MUFA	37.37	35.72	28.63	0.454	<0.001	0.070
PUFA	19.12	21.31	28.59	0.412	<0.001	0.081
UFA	56.49	57.03	57.23	0.647	0.595	0.851
SFA	43.51	42.97	42.77	0.263	0.115	0.302
UFA:SFA	1.30	1.33	1.34	0.016	0.156	0.376

LT, M. longissimus thoracis; UFA, unsaturated fatty acids; SFA, saturated fatty acids; SEM, standard error of the mean; MUFA, monounsaturated fatty acid (the sum C18:1n-9); PUFA, polyunsaturated fatty acid (the sum of C18:2n-6, C18:3n-3, C20:4n-6); UFA, unsaturated fatty acid (the sum of MUFA, PUFA); SFA, saturated fatty acid (the sum of C10:0, C14:0, C16:0, C17:0, C18:0, C20:0).

**Table 6:** Effects of dietary unsaturated to saturated fatty acids ratios on fatty acid composition of subcutaneous adipose (% total fatty acids)

Items	UFA to SFA ratio			SEM (n = 5)	p-value	
	
	1:1	2:1	3:1		Linear	Quadratic
C10:0	0.08	0.09	0.07	0.01	0.297	0.126
C12:0	0.04	0.07	0.05	0.01	0.172	<0.001
C14:1n-9	0.94	1.19	1.00	0.05	0.265	<0.001
C16:0	21.80	23.06	21.21	0.55	0.672	0.080
C16:1n-9	1.41	1.60	1.27	0.07	0.347	0.001
C17:0	0.38	0.38	0.41	0.02	0.060	0.091
C18:0	15.96	13.74	13.17	0.42	0.002	0.432
C18:1n-9	33.00	32.75	31.10	0.63	0.031	0.054
C18:2n-6	23.21	24.17	28.17	0.44	<0.001	0.121
C18:3n-3	0.97	1.08	1.77	0.02	<0.001	0.075
C20:0	0.22	0.18	0.16	0.01	<0.001	0.655
C20:1n-9	0.69	0.52	0.42	0.02	<0.001	0.780
C20:2n-6	0.85	0.82	0.80	0.02	0.147	0.364
C20:3n-6	0.17	0.15	0.16	0.01	0.048	0.013
C20:4n-6	0.27	0.22	0.24	0.02	0.066	0.013
MUFA	36.04	36.06	33.79	0.73	0.103	0.119
PUFA	25.48	26.43	31.14	0.47	<0.001	0.077
UFA	61.52	62.49	64.93	1.01	0.040	0.093
SFA	38.48	37.51	35.07	0.64	0.015	0.299
UFA:SFA	1.60	1.67	1.86	0.04	0.002	0.103

UFA, unsaturated fatty acids; SFA, saturated fatty acids; SEM, standard error of the mean; MUFA, monounsaturated fatty acid (the sum C14:1n-9, C16:1n-9, C18:1n-9, C20:1n-9); PUFA, polyunsaturated fatty acid (the sum of C18:2n-6, C18:3n-3, C20:2n-6, C20:3n-6, C20:4n-6); UFA, unsaturated fatty acid (the sum of MUFA, PUFA); SFA, saturated fatty acid (the sum of C10:0, C12:0, C16:0, C17:0, C18:0, C20:0).

**Table 7:** Effects of dietary unsaturated to saturated fatty acids ratios on meat quality traits of finishing pigs

Items	UFA to SFA ratio			SEM (n = 5)	p-value	
	
	1:1	2:1	3:1		Linear	Quadratic
pH_45 min_	6.41	6.35	6.34	0.02	0.263	0.526
pH_24 h_	5.74	5.69	5.69	0.02	0.097	0.163
Color score	3.67	3.00	2.50	0.08	<0.001	0.704
Shear force (N)	4.43	4.37	4.36	0.02	0.007	0.328
Drip loss (%)	3.65	3.85	3.84	0.06	0.032	0.059
Cooking loss (%)	32.20	32.34	33.43	0.56	0.017	0.103

UFA, unsaturated fatty acids; SFA, saturated fatty acids; SEM, standard error of the mean.
